# Cost-effectiveness analysis of routine immunization and supplementary immunization activity for measles in a health district of Benin

**DOI:** 10.1186/s12962-015-0039-7

**Published:** 2015-08-20

**Authors:** Landry Kaucley, Pierre Levy

**Affiliations:** Health District of Natitingou, Ministry of Health, BP 170, Natitingou, Benin; Paris Dauphine University, LEDa-LEGOS, Place du Marechal de Lattre de Tassigny, 75016 Paris, France

**Keywords:** Measles, Efficiency, Supplementary immunization activity, Routine immunization, Benin

## Abstract

**Background:**

This study was carried out at district level to describe the cost structure and measure the effectiveness of delivering supplementary immunization activity (SIA) and routine immunization (RI) for measles in Benin, a country heavily affected by this disease.

**Methods:**

This cost-effectiveness study was cross sectional and considered 1-year time horizon. RI consists to vaccinate an annual cohort of children aged 0–1 year old and SIA consists to provide a second dose of measles vaccine to children aged 0–5 years old in order to reach both those who did not seroconvert and who were not vaccinated through RI. Ingredients approach to costing was used. Effectiveness indicators included measles vaccine doses used, vaccinated children, measles cases averted and disability adjusted life years averted. Data were collected from all the 18 health care centers of the health district of Natitingou for the year 2011. In the analysis, the coverage was 89 % for RI and 104 % for SIA.

**Results:**

SIA total cost was higher than RI total cost (15,796,560 FCFA versus 9,851,938 FCFA). Personnel and vaccines were the most important cost components for the two strategies. Fuel for cold chain took a non-negligible part of RI total cost (4.03 %) because 83 % of refrigerators were working with kerosene. Cost structures were disproportionate as social mobilization and trainings were not financed during RI contrarily to SIA. In comparison with no intervention, the two strategies combined permitted to avoid 12,671 measles cases or 19,023 DALYs. The benefit of SIA was 5601 measles cases averted and 6955 additional DALYs averted. Cost per vaccinated child for SIA (442 FCFA) was lower than for RI (1242 FCFA), in line with previous data from the literature. Cost per DALY averted was 2271 FCFA (4.73 USD) for SIA and 769 FCFA (1.60 USD) for RI. Analysis showed that low vaccine efficacy decreased the cost-effectiveness ratios for the two strategies. SIA was more cost-effective when the proportion of previously unvaccinated children was higher. For the two strategies, costs per DALY were more likely to vary with measles case fatality ratio.

**Conclusions:**

SIA is costlier than RI. Both SIA and RI for measles are cost-effective interventions to improve health in Benin compared to no vaccination. Policy makers could make RI more efficient if sufficient funds were allocated to communications activities and to staff motivation (trainings, salaries).

**Electronic supplementary material:**

The online version of this article (doi:10.1186/s12962-015-0039-7) contains supplementary material, which is available to authorized users.

## Background

Measles is one of the most serious infectious human diseases as it can cause severe illness, lifelong complications and death. WHO estimated 45 million measles cases and 1.1 million measles related deaths occurring each year in developing countries [1]. Benin is one of the 47 measles high burden countries in the world [2]. To meet measles mortality and morbidity reduction goals, WHO recommended four strategies: (1) strengthening routine immunization to achieve and sustain high coverage, (2) providing a second opportunity for measles immunization, (3) conducting epidemiologic surveillance with high laboratory confirmation of cases and outbreaks and (4) insuring that improved case management is implemented.

Benin is a small country of West Africa (112,622 Km^2^; 7,862,944 inhabitants) [3]. It is a low income country divided in 33 health districts. The health district of Natitingou is located in the north east region of Benin. It is a mountainous region. It covers an area of 3760 Km^2^ with 222,785 inhabitants estimated in 2011 (projection from 2002 national census); most of them are farmers [4]. There are 18 health facilities that provide immunization services.

The first dose of measles containing vaccine (MCV) is given in Benin to children at 9 months through Routine Immunization program (RI). The second opportunity for measles immunization is provided through Supplementary Immunization Activities (SIAs). These SIAs are follow-up campaigns conducted every 3 years since 2001 and targeting children aged 0–59 months. The second opportunity for measles vaccination aims at reaching children who were not vaccinated through RI and to protect children who did not seroconvert with the first dose. By doing so, the population could reach the 95 % population immunity threshold (herd immunity) necessary to stop measles virus circulation. By organizing SIAs campaigns, Benin saw a dramatic fall of measles reported cases with 334 measles cases reported in the first semester of the year 2011 while 5531 cases where annually reported on average during the decade 1991–2000 [5]. RI consists in providing all vaccinations listed in a country immunization program schedule. Services are delivered on an ongoing basis from permanent locations throughout the year. RI usually targets children under 1 year of age. In Benin, the following vaccines are administrated through RI to children under 1 year and to pregnant women: BCG, Pentavalent (DTP-Hep-Hib), OPV, MCV, PCV13, YFV and TT. SIAs are provided from multiple permanent and temporary locations. These campaigns usually have a short duration (1 week) and target children under 5 or 15. RI target population of children aged 0–12 months was estimated to 8911 children and SIA target population was estimated to 34,131 children aged 0–59 months, both for the year 2011. During this year, 7933 children under 1 year old were reached through RI corresponding to a coverage of 89 %. SIA permitted to vaccinate 35,564 children aged 0–59 months corresponding to a coverage of 104 %. A coverage rate of >100 % is commonly observed during SIAs in Benin, usually related to an inaccurate estimation of the target population because the number of eligible children came from not up-to-date national census data.

In a context of global economic crisis and resources scarcity, efficiency of measles immunization strategies needs to be taken into account in African health districts heavily affected by this disease. The operational level needs to know the economic value of their activities as this could help them to get more results at a lesser or equivalent costs. Ignorance of measles immunization costs, neglect of these costs, ignorance of the relative weight of cost components and lack of knowledge of the financial needs cause difficulties in planning, resources mobilization and resources allocation. Nothing is known about the efficiency of SIA and RI for measles in Benin. To fill this gap, this work was initiated by the health district of Natitingou using primary data from routine immunization and measles mass campaign for the year 2011.

The first objective of this paper was to describe cost structure of measles immunization strategies (RI and SIA) in a rural health district. Secondary, this study aimed at measuring the benefits of these strategies on population health. Finally, we assessed the efficiency of each strategy and we compared the cost-effectiveness ratios with a ‘no vaccination’ strategy. Effectiveness indicators took into account not only the immediate results of immunization (vaccine doses used, vaccinated children) but also the impact on final health outcomes (measles cases averted, disability adjusted life years averted). This work will help immunization services providers at health facility level to streamline measles immunization practices for a greater efficiency. The results could also provide guidance to policy makers in Benin for resources allocation.

## Methods

This study was a cross-sectional cost-effectiveness analysis of delivering measles RI and SIA for the year 2011 in the health district of Natitingou. Data were collected retrospectively and the analysis has only considered one-year time horizon. All the 18 health care centers delivering immunization services were included. Data were analyzed by Microsoft Excel software.

RI is a component of the minimum package of activities provided in health facilities in Benin. RI services were provided in the 18 health facilities through two strategies: fixed and outreach strategy. Fixed strategy was planned for children living within less than 5 km from the health center and outreach strategy was planned for children living beyond 5 km from the facility. Health workers planned two fixed strategies sessions and 1 outreach strategy session per week. All health care centers had the same configuration: one dispensary and one maternity ward. Staff consisted of 4 persons: a nurse, a midwife and two caregivers. All staffs were equitably involved in immunization activities. Vaccines were supplied monthly by the district team from the regional storehouse to three secondary storehouses of the district. Health workers were responsible for transporting vaccines from the district storehouses to their respective health facilities by motorcycle. Health facilities were equipped with cold chain materials (refrigerators, cold boxes, vaccines carriers) and motorcycles. Data on the number of vaccinated children were collected on tally sheets at each immunization session. A synthesis was made at the end of the month by the head of the health center before transmission to district team.

The Ministry of Health planned SIA for measles to vaccinate children unreached through RI and to enhance immunity of children remained susceptible despite the first dose of vaccine received. A micro planning was done at district level taking into account human, logistical and financial needs. The strategy adopted was vaccination at fixed locations to which parents should bring children. A total of 29 vaccination posts were set up. Vaccination team at a station consisted of 3 persons (2 health workers and 1 volunteer) and should vaccinate at least 250 children per day. Supervision of vaccination teams was ensured by 6 supervisors who should supervise each 6 teams per day. Supervisors and vaccinators had been trained during a 1-day training session. Communication activities were strengthened with involvement of town criers.

### Measuring costs

Costs taken into account included recurrent and capital costs. Both economic and financial costs were considered. Some costs were specific to measles immunization and others were shared with others activities. Data for SIA specific operational costs came from the campaign financial reports and micro planning tools. Shared costs for measles RI activities were supposed to represent 9 % of RI delivery total costs; as measles vaccine was one of the 11 antigens administrated in Benin RI schedule. RI recurrent costs were analyzed using ingredients approach to costing. Data on vaccines used, consumables and management tools used were empirically obtained at health facilities level from vaccine usage reports, tally sheets, vaccine management databases. Costs for personnel, transport, supervision, trainings, fuel for cold chain, electricity and maintenance were obtained from interviews, questionnaires, direct surface measurement of buildings and exploitation of documents (administrative and financial reports, log books), both at health facilities and district administration level. Costs were expressed in local currency (FCFA) and in USD, using a fixed exchange rate of 480 FCFA for 1 USD (2011 average exchange rate from http://oanda.com).

Recurrent costs components included: vaccines, consumables, management tools, personnel, transport, maintenance, supervision, trainings, social mobilization, fuel for cold chain materials functioning and electricity bills:

- Vaccines costs were calculated by multiplying the cost per dose of MCV by the number of doses used. MCV dose unit price was 0.24 USD, derived from National EPI vaccine’s purchase orders.
- Consumables included syringes (auto disable and dilution), safety boxes and cotton. Cost per unit of syringes and safety boxes were derived from WHO’s Pre Qualify Standard (PQS) devices catalogue [6]. The amount of medical accessories used was estimated on the basis of immunization management report. The same method was used to estimate the cost of management tools (data collection spreadsheets, registers).
- Personnel costs were defined by wages and allowances paid to the staff involved in immunization activities. They were calculated by exploitation of staff payment vouchers and other documents considering the estimated proportion of time spent for immunization activities. Staff devoted 2 days per week to fixed strategy sessions and 1 day per week to outreach strategy sessions. A fixed strategy session took 4 h corresponding to half of a daily working time. An outreach strategy session took all the daily working time, as vaccination teams had to travel more than 5 km before reaching the village were children should be vaccinated. Roads in Natitingou were bad and such a distance could take one to 2 h travelling by motorcycle.
- Transport was related to expenditures for vaccine supply and transportation fees for outreach strategies. These costs were obtained multiplying the distance covered by the average fuel consumption of the vehicle used and by the unit price of a liter of fuel. From exploitation of vehicle user’s manuals, average fuel consumption considered was 16 L/100 km for cars and 2 L/100 km for motorcycles. The unit price of a liter of fuel was 625 FCFA for the year 2011 in the health district of Natitingou.
- Maintenance included costs for cold chain, cars and motorcycles reparation and periodic maintenance. For cars and motorcycles, this shared cost was estimated applying the proportion of kilometers devoted to immunization activities. Log books available in cars allowed to estimate kilometers covered. For motorcycles, distances covered were estimated from interviews with staff conducting immunization activities.
- Supervision included all costs related to supportive supervision (per diems, fuel, reproducing management tools). Data for this cost component came from exploitation of district administrative and financial reports.
- Trainings corresponded to total expenditures for trainings organized for SIA or RI (venue hiring charges, training materials, per diems). No training was organized as part of RI. Data for SIA came from exploitation of the campaign financial reports available at district administration level.
- Social mobilization was related to cost of communication activities (advocacy meetings, banners, news release). No expense was performed for social mobilization in RI. Data for SIA came from exploitation of the campaign financial reports available at district administration level.
- Fuel for cold chain corresponded to total cost of kerosene used in health facilities where cold chain materials were working with kerosene. There were 18 refrigerators and 3 freezers used for immunization activities in the health district. The 15 kerosene-functioning refrigerators were all of the same type (RCW50 EK) and consumed on average 35 L of kerosene per month. The unit price of a liter of kerosene was 700 FCFA for the year 2011 in the health district of Natitingou.
- Electricity was related to the shared cost of electricity for the 3 health care centers where cold chain materials were working with electricity. This cost component was calculated multiplying the kilowatt-hours (kWh) consumption of the cold chain material used by the unit price of a kWh fixed by the national electricity company. Energy consumption of cold chain equipment were derived from WHO’s PQS devices catalogue [6]: 2.35 kWh per 24 h for freezers and 0.61 kWh per 24 h for refrigerators. Electricity Company pricing was 125 FCFA per kWh.

Using linear amortization method, capital costs for buildings, motorcycles, cars and cold chain materials were estimated by dividing their respective cost by the useful life time. In the case of SIA, this was later divided by 0.019 (7 days of campaign over 365 days of the year). The result obtained was later multiplied first by the proportion of use for immunization activities and secondary by the estimated proportion for measles immunization. Both time and space were considered for buildings (number of days used a week and proportion of surface used). The share of kilometer attributable to immunization activities was considered for cars and motorcycles. Useful life time considered was 50 years for buildings, 10 years for refrigerators, freezers, cold boxes and vaccine carriers, 5 years for cars and 3 years motorcycles following the amortization plan of the health district.

### Calculating effectiveness

Four effectiveness indicators were estimated: MCV doses used and vaccinated children relate to productive efficiency while the number of measles cases averted and disability adjusted life years averted refer to allocative efficiency. The primary effectiveness indicator selected was disability adjusted life years (DALYs) averted. It is a widely used indicator that allows easy comparison with a ‘no vaccination’ strategy and with others public health interventions.

MCV doses used represents the amount of vaccines used per strategy. This was empirically obtained from district vaccine management tools (vaccine usage reports, tally sheets, vaccine management databases). Vaccinated children corresponds to the total of targeted children that received a shot, having in mind that for RI the target population is defined by children under 1 year whereas the target population for SIA extends to children under 5 years. Measles cases averted depend on both the number of vaccinated persons and vaccine efficacy. MCV efficacy after a single dose depends on the age of vaccination: 85 % of children seroconvert after receiving a shot when vaccination is done at the age of 9–11 months whereas after 1 year of age, vaccine efficacy raises to 95 % [7]. To facilitate comparison of final outcomes with a ‘no vaccination’, we estimated DALYs averted. DALY’s calculation is explained more in depth in the following section.

### Disability adjusted life years

Immunization with MCV enables to increase the target population well-being experienced by averting deaths, and by averting sufferings due to measles. The concept of disability adjusted life years, as routinely used by WHO, is a useful measure of the burden of measles on population of interest. DALYs for a specific cause are calculated as the sum of the years of life lost due to premature death (YLL) from that cause and the years of healthy life lost as a result of disability (YLD) for incident cases of the health condition as follows: *DALY* = *YLL* + *YLD* [8, 9].

**Fig. 1:**
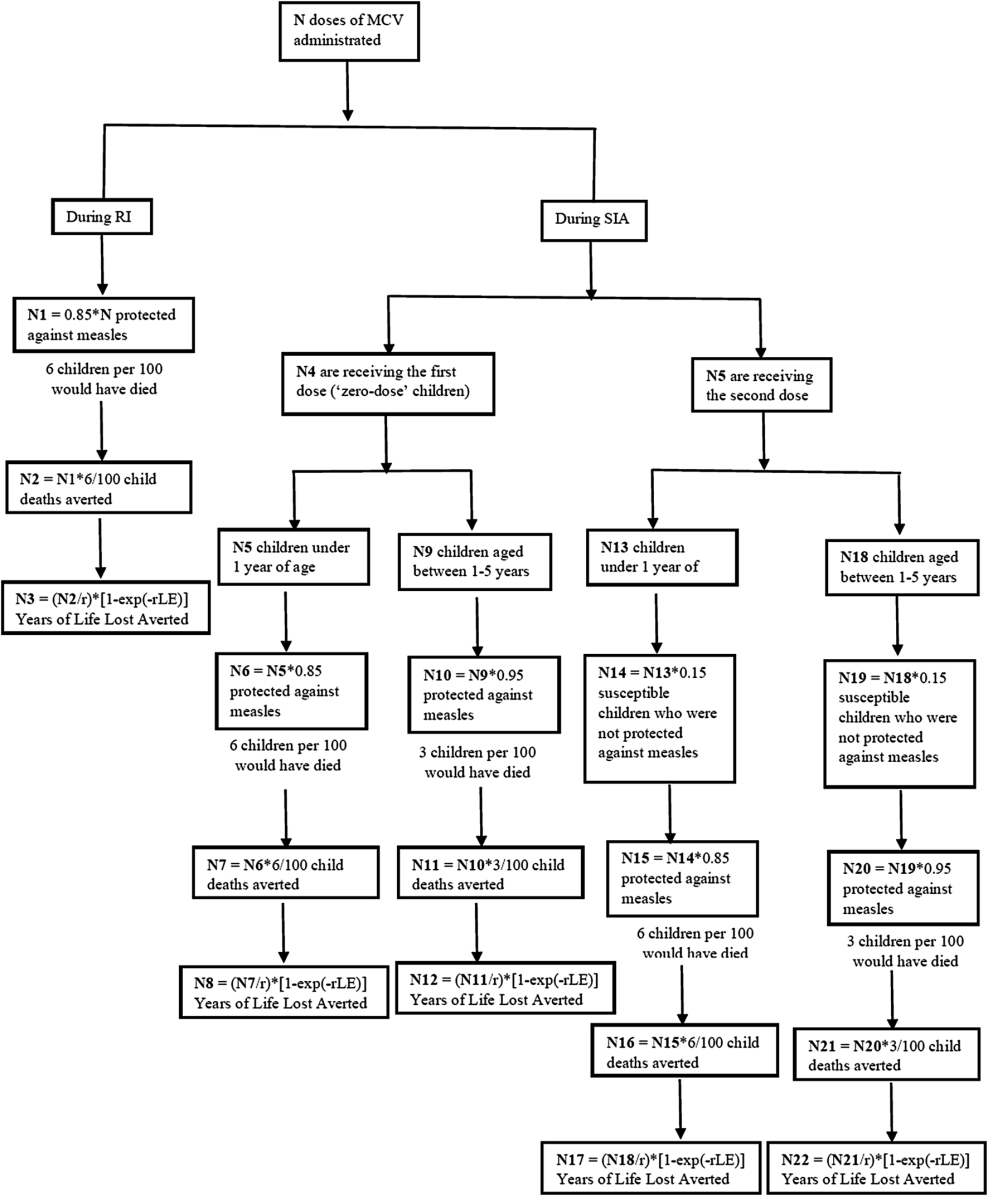
Diagram used to estimate the years of life lost

YLL formula is: (*N/r*) × [1 − *exp*(−*rLE*)], where N is the number of deaths; LE is standard life expectancy at age of death and r is the discount rate. Measles deaths averted are estimated on the basis of the number of effectively immunized children after being vaccinated and death rate from measles related cause. The number of effectively immunized children depends on vaccine efficacy [7]. Measles case fatality ratios were age specific: 6 % at 6–11 months and 3 % at 1–5 years [10, 11]. Standard life expectancy at birth in Benin is set as 52.5 years [12]. The discount rate used was 3 % in accordance with WHO recommendations. DALYs were calculated without age weighting. Figure 1 shows the diagram used to estimate YLL from doses of administrated vaccine per strategy.

The formula for YLD calculation is: *YLD* = *∑ I* × *DW* × [1 *−* *exp*(−*rL*)]/*r*, where I is the number of incident cases; DW is the disability weight; L is the average duration of disability until remission or death and r is the discount rate. The incident cases were episodes of measles case and the classic complications associated with measles (diarrhea, pneumonia, otitis media, corneal scarring, encephalitis and subacute sclerosing pan encephalitis). Diarrhea is reported in 8 % of measles cases [13]. Six per cent of measles cases are complicated by pneumonia and corneal scarring [13]. Otitis media occurs in about 7 % of cases [13]. About 1 child in every 1000 who get measles will develop encephalitis [13]. Subacute sclerosing pan encephalitis (SSE) is a rare degenerative central nervous system disease that occurs on average in 8 per 1,000,000 reported measles cases [13]. Duration and disease disability weights relevant for incident cases were extracted from the Global Burden of Diseases study [14]: measles episode (2 weeks, 0.152); diarrhea (1 week, 0.105); pneumonia (2 weeks, 0.373); otitis media (2 years, 0.023); corneal scarring (34.8 years, 0.277); encephalitis (34.8 years, 0.616); SSE (36.7 years, 0.666).

### Sensitivity analysis

A one-way sensitivity analysis was used to test the robustness of the initial results on the cost per DALY averted with alternatives values of key parameters. We explored the variation of some cost components for RI: vaccines cost, staff cost, supervision cost, trainings and communication costs. Vaccine unit dose price variation assumed was ±50 %. We explored effects of twofold increase of the base-case staff cost. Increasing supervision costs by multiplying the supervision frequency from 4 to 6 per annum was tested. Effects of adding trainings and communication costs in RI, the same amount as SIA base-case, was analyzed. To compare our results with studies which used only recurrent cost components, a sensitivity analysis was conducted to explore effects of excluding capital costs from the base-case analysis. As vaccine efficacy and measles CFR are two key parameters influencing the number of measles cases averted, we performed a sensitivity analysis for these two variables. A measles post-outbreak survey conducted in Democratic Republic of Congo shows that measles vaccine efficacy could fall to 60 % [15]. Higher measles CFRs are observed during outbreaks, among under 5 years old children, in cases with complications and among children living in rural areas [16]. A review of 25 community-based studies conducted in India shows that measles CFRs vary between 0.00 and 31.25 % [11]. As vaccination coverage affects both RI and SIA effectiveness, a sensitivity analysis was performed for this variable. For RI, coverage variation used was 50–98 %, based on extreme levels achieved in the past years after analysis of the health district immunization databases. For SIA, lowest coverage used was 80 %, based on coverage achieved by the health district during the past SIAs. Because the proportion of previously unvaccinated children during SIA (‘zero-dose’ children) is a parameter that allows to assess the benefit of SIA, we performed a sensitivity analysis on the percentage of children reached with the second dose. We assumed 0–50 % variation of ‘zero dose’ children respecting the age groups proportion of the base-case. Table 1 shows the base-case and alternatives values of parameters used in the sensitivity analysis. Results were presented in tornado diagrams.

**Table 1:** Base-case and alternative values of parameters used in the sensitivity analysis

	Variations tested	Base case values	Alternatives values
Routine immunization	Vaccines cost (MCV unit dose price)	120 FCFA/Dose	60–180 FCFA/Dose
	Supervision cost: Increasing supervision frequency	4 supervisions per annum	6 supervisions per annum
	Personnel cost : increasing total personnel cost	3,599,536 FCFA	7,199,072 FCFA
	Inclusion of communication costs	0	371,000 FCFA
	Exclusion of capital costs	14,748,858 FCFA	0
	Vaccine efficacy	85 %	60–99 %
	Measles CFR	6 %	0.00–31.25 %
	RI coverage	89 %	50–98 %
Supplementary immunization activity	Vaccines cost (MCV unit dose price)	120 FCFA/Dose	60–180 FCFA/Dose
	Exclusion of capital costs	279,694 FCFA	0
	Proportion of unvaccinated children (‘zero-dose’)	2 %	0–50 %
	Vaccine efficacy	85 %	60–99 %
	Measles CFR	6 %	0–31.25 %
	SIA coverage	104 %	80–104 %

## Results

### Costs

Overall, 25,648,498 FCFA (53,434.37 USD) were spent to protect children against measles in the health district of Natitingou in 2011 (RI and SIA) (Table 2). SIA total cost was higher than RI total cost with respectively 15,796,560 FCFA (32,909.50 USD) and 9,851,938 FCFA (20,524.87 USD). Recurrent costs took the larger cost share for the two strategies: 62.51 % of total cost for RI and 99.56 % of total cost for SIA. Personnel, and vaccines were the most important recurrent cost components for RI representing respectively 36.54 and 11.35 % of total cost. They were followed by fuel for cold chain material (4.03 %) and consumables (3.97 %). Although important, social mobilization and trainings were not financed in RI. For SIA, the three important recurrent cost components were personnel (30.07 %), vaccines (29.93 %) and consumables (22.83 %). The larger cost share of vaccines in SIA is due to a larger target population of children up to 5 years of age whereas RI is only devoted to children under 1 year. It is to be noticed that per diems paid to the staff during SIA were higher than the total adjusted wages and allowances they received during 1 year.

**Table 2:** Costs by component for measles RI and SIA in the health district of Natitingou in 2011

Cost components	RI		SIA		Overall (RI + SIA)	
	Amount (FCFA)	%	Amount (FCFA)	%	Amount (FCFA)	%
Reccurent costs						
Vaccines	1,118,400	11.35	4,728,000	29.93	5,846,400	22.79
Consumables	391,161	3.97	3,606,188	22.83	3,997,350	15.59
Personnel	3,599,536	36.54	4,750,000	30.07	8,349,536	32.55
Transport	95,258	0.97	625,436	3.96	720,694	2.81
Communication	0	0.00	371,000	2.35	371,000	1.45
Trainings	0	0.00	454,000	2.87	454,000	1.77
Supervision	220,579	2.24	830,550	5.26	1,051,129	4.10
Maintenance	98,010	0.99	0	0.00	98,010	0.38
Wastage management	58,501	0.59	137,500	0.87	196,001	0.76
Surveillance	143,150	1.45	216,000	1.37	359,150	1.40
Fuel for cold chain	396,900	4.03	7,541	0.05	404,441	1.58
Electricity	36,464	0.37	693	0.00	37,156	0.14
Capital costs						
Buildings	1,188,000	12.06	22,572	0.14	1,210,572	4.72
Cars and motorcycles	1,120,320	11.37	21,286	0.13	1,141,606	4.45
Cold chain materials	1,385,658	14.06	25,793	0.16	1,411,451	5.50
Total	9,851,938	100	15,796,560	100	25,648,498	100

### Effectiveness

SIA was more effective when referring to the number of vaccines used and the number of vaccinated children as it led to vaccinate five times more children than RI (Table 3). There were 742 previously unvaccinated children (‘zero-dose’ children) among the 35,564 children vaccinated during SIA corresponding to 2 % of total target. More vaccines were wasted during RI (1387 wasted doses) than SIA (1106 wasted doses). Vaccine wastage rate (as defined by the number of doses used minus vaccinated targets over number of doses used) was lower for SIA (3 %) than RI (15 %). To the contrary, RI permitted to avoid more measles cases, measles deaths and DALYs than SIA (Table 4). The benefits of SIA was 5601 measles cases averted, 185 measles death averted and 6955 additional DALYs averted.

**Table 3:** Vaccine doses used, vaccinated children and wasted doses of measles RI and SIA in the health district of Natitingou in 2011

	RI	SIA					Overall (RI + SIA)
		Children receiving first dose		Children receiving second dose		Total SIA	
		<1 year	≥1 year	<1 year	≥1 year		
Vaccine doses used	9320	360	400	2110	33,800	36,670	45,990
Vaccinated children	7933	349	393	2092	32,730	35,564	43,497
Wasted doses	1387	11	7	18	1070	1106	2493

**Table 4:** Measles cases averted, measles deaths averted and DALYs averted for RI and SIA in the health district of Natitingou in 2011

	RI	SIA	Overall (RI + SIA)
Measles cases averted	6743	5601	12,344
Measles deaths averted	405	185	590
DALYs averted	12,815	6955	19,770

### Average costs

Overall, the mean cost per vaccinated child was 590 FCFA (1 USD) but it was 1057 FCFA (2.20 USD) for RI and 431 FCFA (0.90 USD) for SIA (Table 5). SIA had the best cost effectiveness ratios when referring to process outcomes whether considering the cost per MCV dose used or the cost per vaccinated child. In comparison with ‘no vaccination’, cost/DALY averted for RI was 769 FCFA (1.60 USD). The marginal cost was 2271 FCFA (4.73 USD) per supplementary DALY averted for SIA. The two strategies put together had a cost/DALY averted of 1297 FCFA (3 USD).

**Table 5:** Average costs for measles RI and SIA in the health district of Natitingou in 2011

	RI		SIA		Overall (SIA + RI)	
	FCFA	USD	FCFA	USD	FCFA	USD
Cost per dose used	1057	2.20	431	0.90	558	1
Cost per vaccinated child	1242	2.59	442	0.92	590	1
Cost per measles case averted	1461	3.04	2820	5.88	2078	4
Cost per measles death averted	24,351	50.73	85,422	177.96	43,508	91
Cost per DALY averted	769	1.60	2271	4.73	1297	3

**Fig. 2:**
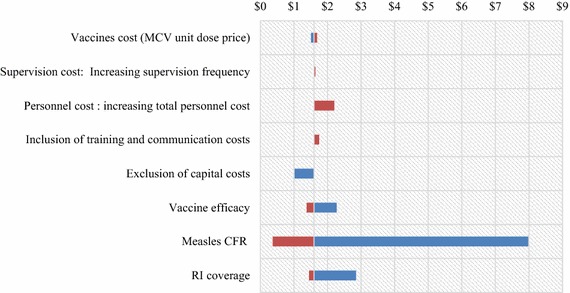
Tornado diagram showing the range of cost/DALY averted for routine immunization (RI). The vertical line between $1 and $2 represents the base-case cost/DALY of RI compared to no intervention, along with the specific value calculated. The widths of the *bars* show the variation in cost/DALY as each parameter is varied from *lower* bound to *upper* bound. *$* USD

**Fig. 3:**
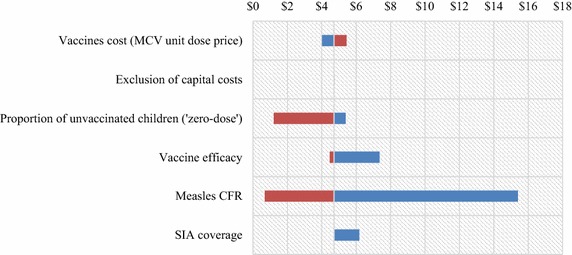
Tornado diagram showing the range of cost/DALY averted for supplementary immunization activity (SIA). The *vertical line* between $4 and $6 represents the base-case cost/DALY of SIA compared to no intervention, along with the specific value calculated. The widths of the *bars* show the variation in cost/DALY as each parameter is varied from *lower* bound to *upper* bound. *$* USD

In the sensitivity analysis, the cost per DALY for RI varied from 0.37 to 7.98 USD (Fig. 2) and from 0.69 to 15.40 USD for SIA (Fig. 3). In both cases, cost per DALY was more sensitive to changes in measles CFR. Reducing measles CFR from 31.25 to 0.00 % lead to an increase of the cost per DALY averted from 0.37 to 7.98 USD for RI and from 0.69 to 15.40 USD for SIA. Exclusion of capital costs did not affect significantly the cost/DALY for the two strategies. However the analysis showed that cost-effectiveness ratios of the two strategies varied significantly following vaccine coverage and vaccine efficacy. High vaccine coverage and high measles vaccine efficacy improve the cost-effectiveness of the two interventions. With a twofold increase of the base-case personnel cost, the cost/DALY increased from 1.6 to 2.2 USD. In the same manner, when trainings and communication costs were included in the base-case analysis of RI, the cost/DALY only increased from 1.5 to 1.75 USD. During SIA, when the proportion of ‘zero-dose’ children vary from 0 to 50 %, the cost/DALY decrease from 5.39 to 1.21 USD.

## Discussion

### Regarding costs

Results from the present study are in line with previous studies on the fact that SIAs are costlier than RI [17, 18]. In this study, total cost of SIA for measles was 15,796,560 FCFA (32,909.50 USD) versus 9,851,938 FCFA (20,524.87 USD) for RI. Personnel and vaccines costs took the large part of the two strategies total cost (respectively 36.54 and 11.35 % for RI; 30.07 and 29.93 % for SIA). ‘Personnel’ is usually cited among the highest cost components in immunization costs analysis [18–21]. In the context of Natitingou, close to that described in a Cameroonian health district [21], medical staff was poorly paid. The highest wage for a health professional involved in immunization activities was 100,000 FCFA (208 USD) per month corresponding to 7 USD per day. During SIA, medical staff was paid 7000 FCFA (15 USD) per day. In addition, during SIA, community health workers were recruited and paid 5000 FCFA (10 USD) per day to support health professionals whereas they did not receive any incentives for their involvement in RI activities. This explains why personnel costs of SIA were higher than that for RI. In addition, savings were made in RI because social mobilization and staff trainings were not financed. A particular attention should be paid to the part took by fuel for cold chain functioning. With 4.03 % of RI total cost, it represents the third cost component behind personnel and vaccines. In fact, 83 % of refrigerators used for immunization activities were working with kerosene. Given, following WHO recommendations, vaccines needs to be permanently stored at health facility level at a temperature between +2 and +8 °C, kerosene supply was a requisite and incompressible expenditure. The use of cold chain material working with solar energy in settings without electricity as alternative should be explored by policy makers in Benin. Savings could be possible with this material, as it requires no fuel for functioning and there is no need of regular spare parts replacement (burners, wicks, glasses) unlike kerosene-functioning refrigerators.

### Regarding effectiveness

RI permitted to vaccinate 89 % of the target population, under the 95 % recommended by the national immunization program. In contrast, SIA reached 104 % of the target in short time of period (7 days). It permitted to vaccinate 35,564 under-five children and the benefit was 5601 supplementary measles cases averted converted to 6955 additional DALYs averted. SIA permitted to save 185 children who should have probably died from measles despite RI. This strength of SIA to reach usually unreachable children through RI make it an intervention that contributes to equity in health and a preferable platform to deliver second dose of MCV in settings where RI is not effective enough [16, 22, 23]. Fewer vaccines were lost during SIA compared to RI (3 % of vaccine wastage rate versus 15 %). It could be the direct consequence of non-financing social mobilization activities in RI. Yet, social mobilization contributes to encourage and motivate the public to fully participate in immunization activities. Thus, it helps to gather a lot of targets at vaccination location and then lowers vaccine wastage by applying open vial policy for lyophilized vaccines (MCV should be used within 6 h after the vial being opened).

### Regarding efficiency

In accordance with estimations in Niger (190 FCFA for SIA and 573 FCFA for RI) [17] and Uganda (0.58 USD for SIA and 1 USD for RI) [24], the cost per vaccinated child in this study was lower for SIA than RI (431 FCFA versus 1051 FCFA). SIA cost per vaccinated child was similar to findings for poliomyelitis mass campaigns conducted in West Africa [18, 20, 25]. SIA had better productive efficiency indicators because more children were reached and fewer vaccines were wasted. To improve RI productive efficiency, more political and community implication should be conjugated to higher health professionals’ motivation, media and local leaders’ mobilization. Unfortunately, as shown by cost structures’ analysis, financing communication activities and staff trainings was neglected by the health district.

Whatever is the strategy used, measles immunization is known to be a cost-effective intervention [23, 24]. In our study, in comparison with no intervention, cost per DALY averted was 2271 FCFA (4.73 USD) for SIA; 769 FCFA (1.60 USD) for RI and 1297 FCFA (3 USD) for both strategies combined. These cost-effectiveness ratios are quite under those of interventions known to be the most efficient interventions in Afro-E, a WHO defined region comprising countries in sub-Sahara Africa with high child mortality [26]. Analysis showed that cost per DALY averted never exceeded 16 USD whatever the possible extreme variations of chosen parameters were. The cost per DALY was higher, but still under 16 USD, when measles CFR was very low (0.00 %). But it seems to be an extreme scenario as it is far to be the case in the health district of Natitingou and most of health districts of Benin, where malnutrition, vitamin A deficiency, poor cases management and intercurrent infections like malaria and HIV contribute to high measles fatality rate. A twofold increase of personnel costs did not impact negatively the cost/DALY for RI neither was inclusion of trainings and communication costs in the base-case analysis. Therefore, there is no need to make savings on these cost components. In contrary, allocating funds to this cost components would increase the attention of health professionals to RI activities and contribute indirectly to a greater efficiency. SIA was more cost-effective when the proportion of previously unvaccinated was higher. In other words, more RI failed to vaccinate targeted children, more SIA became a cost-effective intervention.

### Implications for decision-making

Since non-financing communication activities is prejudicial to RI effectiveness, the health district managers should plan and finance annually social mobilization activities for RI. District administration needs also to organize short-term trainings for health professionals as it will strengthen their capacity in conducting vaccination activities and it can increase their motivation. There is a need for immunization policy makers in Benin to think about adopting low-cost cold chain materials in terms of energy consumption. As discussed above, there is possibility to make savings with solar system materials. Importantly, vaccine efficacy should be maintained at its highest level to improve the efficiency of both RI and SIA. In other words, vaccine should be properly stored and transported respecting the recommended temperature at all steps of the supply chain. Measles vaccine is very sensitive to heat and rapidly lost its efficacy when exposed to temperature over +8 °C for a long time. Usually, breaks in cold chain occurs at health facility level or at vaccination delivery point where health professionals fail to keep the vaccine in the right temperature because of poor cold chain materials maintenance.

Health districts in Benin are organized in the same way. Staffing and resources for immunization activities are allocated in the same manner. Moreover, vaccination strategies are the same and measles epidemiologic profile is not different from one health district to another. Accordingly, results from this study and the recommendations derived from it could be transposed to any other health district of the country. In the same way, the results could be useful to immunization policy makers in other sub-Saharan African countries with similar measles disease burden and with the same health system organization.

### Limitations

The use of primary data empirically obtained from the field is a strength of this study. Moreover, this study estimated the costs of delivering one single antigen among those administrated through RI, an unusual case in the literature. However, the study has some limitations. Transport and maintenance costs for RI may have been inaccurately estimated because distances covered by motorcycles were based on health professionals’ statements. Measles mortality may have been inaccurately estimated because we did not used measles case fatality rate in Benin due to lack of accurate data. Measles case fatality rates used came from published data for countries with a similar epidemiological profile to Benin regarding measles. Vaccine coverage should be interpreted carefully because denominators used (targeted children) came from an old national census. There are shortcomings of relying on old GBD data although GBD-2010 data were available [27]. The comprehensive sensitivity analysis conducted with large ranges of values helped to address these uncertainties (Additional file 1).

## Conclusion

Key findings from this operational research, the first to be conducted on the costs and effectiveness of measles vaccination strategies in Benin, could be summarized as followed:

- First, SIA for measles cost more than RI for measles. However, costs structure of the two strategies was disproportionate.
- Second, SIA and RI for measles are cost-effective interventions to improve health in Benin compared to no vaccination. The proportion of ‘zero-dose’ children affects significantly SIA cost-effectiveness. A twofold increase of personnel costs or inclusion of trainings and communication costs in the base-case analysis do not impact negatively the cost/DALY for RI.

Benin have set measles elimination goal for the year 2020. It is possible to meet this goal if sufficient funds are mobilized and efficiency is taken into account. This work address the question of the costs, the effectiveness and the efficiency of delivering two dose of measles vaccination combining RI and SIA at district level in Benin. Future studies should be focused on measles immunization activities financing to identify how to ensure sustainable funding.

## Additional file

**Additional file 1.** Excel calculation baseline showing in details how costs and effectiveness indicators were calculated and how tornado diagrams were drawn

## Abbreviations

BCG: Bacille de Calmette et Guérin
CER: cost effectiveness ratio
CFR: case fatality ratio
DALY: disability adjusted life year
DTP-Hep-Hib: diphteria, tetanus, pertussis, hepatitis B and *Heamophilus Influenza* type B vaccine
DW: disability weight
EPI: Expended Program on Immunization
FCFA: Franc de la Communauté Française d’Afrique
HIV: human immunodeficiency virus
Km: kilometer
kWh: kilowatt-hours
LE: life expectancy
MCV: measles containing vaccine
OPV: oral polio vaccine
PQS: Pre Qualify Standard
RI: routine immunization
SIA: supplementary immunization activity
SSE: subacute sclerosing pan encephalitis
TT: tetanus toxoid
USD: United States Dollar
WHO: World Health Organization
YFV: yellow fever vaccine
YLD: years of life disability
YLL: years of life lost
